# The design, implementation, and evaluation of a blended (in-person and virtual) Clinical Competency Examination for final-year nursing students

**DOI:** 10.1186/s12909-024-05935-9

**Published:** 2024-08-28

**Authors:** Rita Mojtahedzadeh, Tahereh Toulabi, Aeen Mohammadi

**Affiliations:** 1 Department of E-Learning in Medical Education, Center of Excellence for E-learning in Medical Education, School of Medicine, Tehran University of Medical Sciences, Tehran, Iran; 2 Department of Medical Education, Smart University of Medical Sciences, Tehran, Iran; 3 Cardiovascular Research Center, School of Nursing and Midwifery, Lorestan University of Medical Sciences, Khorramabad, Iran

**Keywords:** Clinical Competency Examination (CCE), Objective Structural Clinical Examination (OSCE), Blended method, In-person, Virtual, Satisfaction, Anxiety

## Abstract

**Introduction:**

Studies have reported different results of evaluation methods of clinical competency tests. Therefore, this study aimed to design, implement, and evaluate a blended (in-person and virtual) Competency Examination for final-year Nursing Students.

**Methods:**

This interventional study was conducted in two semesters of 2020–2021 using an educational action research method in the nursing and midwifery faculty. Thirteen faculty members and 84 final-year nursing students were included in the study using a census method. Eight programs and related activities were designed and conducted during the examination process. Students completed the Spielberger Anxiety Inventory before the examination, and both faculty members and students completed the Acceptance and Satisfaction questionnaire.

**Findings:**

The results of the analysis of focused group discussions and reflections indicated that the virtual CCE was not capable of adequately assessing clinical skills. Therefore, it was decided that the CCE for final-year nursing students would be conducted using a blended method. The activities required for performing the examination were designed and implemented based on action plans. Anxiety and satisfaction were also evaluated as outcomes of the study. There was no statistically significant difference in overt, covert, and overall anxiety scores between the in-person and virtual sections of the examination (*p* > 0.05). The mean (SD) acceptance and satisfaction scores for students in virtual, in-person, and blended sections were 25.49 (4.73), 27.60 (4.70), and 25.57 (4.97), respectively, out of 30 points, in which there was a significant increase in the in-person section compared to the other sections. (*p* = 0.008). The mean acceptance and satisfaction scores for faculty members were 30.31 (4.47) in the virtual, 29.86 (3.94) in the in-person, and 30.00 (4.16) out of 33 in the blended, and there was no significant difference between the three sections (*p* = 0.864).

**Conclusion:**

Evaluating nursing students’ clinical competency using a blended method was implemented and solved the problem of students’ graduation. Therefore, it is suggested that the blended method be used instead of traditional in-person or entirely virtual exams in epidemics or based on conditions, facilities, and human resources. Also, the use of patient simulation, virtual reality, and the development of necessary virtual and in-person training infrastructure for students is recommended for future research. Furthermore, considering that the acceptance of traditional in-person exams among students is higher, it is necessary to develop virtual teaching strategies.

## Introduction

The primary mission of the nursing profession is to educate competent, capable, and qualified nurses with the necessary knowledge and skills to provide quality nursing care to preserve and improve the community’s health [1]. Clinical education is one of the most essential and fundamental components of nursing education, in which students gain clinical experience by interacting with actual patients and addressing real problems. Therefore, assessing clinical skills is very challenging. The main goal of educational evaluation is to improve, ensure, and enhance the quality of the academic program. In this regard, evaluating learners’ performance is one of the critical and sensitive aspects of the teaching and learning process. It is considered one of the fundamental elements of the educational program [2]. The study area is educational evaluation.

Various methods are used to evaluate nursing students. The Objective Structured Clinical Examination (OSCE) is a valid and reliable method for assessing clinical competence [1, 2]. In the last twenty years, the use of OSCE has increased significantly in evaluating medical and paramedical students to overcome the limitations of traditional practical evaluation systems [3, 4]. The advantages of this method include providing rapid feedback, uniformity for all examinees, and providing conditions close to reality. However, the time-consuming nature and the need for a lot of personnel and equipment are some disadvantages of OSCE [5, 6]. Additionally, some studies have shown that this method is anxiety-provoking for some students and, due to time constraints, being observed by the evaluator and other factors can cause dissatisfaction among students [7, 8].

However, some studies have also reported that this method is not only not associated with high levels of stress among students [9] but also has higher satisfaction than traditional evaluation methods [4]. In addition, during the COVID-19 pandemic, problems such as overcrowding and student quarantine during the exam have arisen. Therefore, reducing time and costs, eliminating or reducing the tiring quarantine time, optimizing the exam, utilizing all facilities for simulating the clinical environment, using innovative methods for conducting the exam, reducing stress, increasing satisfaction, and ultimately preventing the transmission of COVID-19 are significant problems that need to be further investigated.

Studies show that using virtual space as an alternative solution is strongly felt [10–12]. In the fall of 2009, following the outbreak of H1N1, educational classes in the United States were held virtually [13]. Also, in 2005, during Hurricane Katrina, 27 universities in the Gulf of Texas used emergency virtual education and evaluation [14].

One of the challenges faced by healthcare providers in Iran, like most countries in the world, especially during the COVID-19 outbreak, was the shortage of nursing staff [15, 16]. Also, in evaluating and conducting CCE for final-year students and subsequent job seekers in the Clinical Skills Center, problems such as student overcrowding and the need for quarantine during the implementation of OSCE existed. This problem has been reported not only for us but also in other countries [17]. The intelligent use of technology can solve many of these problems. Therefore, almost all educational institutions have quickly started changing their policies’ paradigms to introduce online teaching and evaluation methods [18, 19].

During the COVID-19 pandemic, for the first time, this exam was held virtually in our school. However, feedback from professors and students and the experiences of researchers have shown that the virtual exam can only partially evaluate clinical and practical skills in some stations, such as basic skills, resuscitation, and pediatrics [20].

Additionally, using OSCE in skills assessment facilitates the evaluation of psychological-motor knowledge and attitudes and helps identify strengths and weaknesses [21]. Clinical competency is a combination of theoretical knowledge and clinical skills. Therefore, using an effective blended method focusing on the quality and safety of healthcare that measures students’ clinical skills and theoretical expertise more accurately in both in-person and virtual environments is essential. The participation of students, professors, managers, education and training staff, and the Clinical Skills Center was necessary to achieve this important and inevitable goal. Therefore, the Clinical Competency Examination (CCE) for nursing students in our nursing and midwifery school was held in the form of an educational action research process to design, implement, and evaluate a blended method. Implementing this process during the COVID-19 pandemic, when it was impossible to hold an utterly in-person exam, helped improve the quality of the exam and address its limitations and weaknesses while providing the necessary evaluation for students.

The innovation of this research lies in evaluating the clinical competency of final-year nursing students using a blended method that focuses on clinical and practical aspects. In the searches conducted, only a few studies have been done on virtual exams and simulations, and a similar study using a blended method was not found.

The research investigates the scientific and clinical abilities of nursing students through the clinical competency exam. This exam, traditionally administered in person, is a crucial milestone for final-year nursing students, marking their readiness for graduation. However, the unforeseen circumstances of the COVID-19 pandemic and the resulting restrictions rendered in-person exams impractical in 2020. This necessitated a swift and significant transition to an online format, a decision that has profound implications for the future of nursing education. While the adoption of online assessment was a necessary step to ensure student graduation and address the nursing workforce shortage during the pandemic, it was not without its challenges. The accurate assessment of clinical skills, such as dressing and CPR, proved to be a significant hurdle. This underscored the urgent need for a change in the exam format, prompting a deeper exploration of innovative solutions.

To address these problems, the research was conducted collaboratively with stakeholders, considering the context and necessity for change in exam administration. Employing an Action Research (AR) approach, a blend of online and in-person exam modalities was adopted. Necessary changes were implemented through a cyclic process involving problem identification, program design, implementation, reflection, and continuous evaluation.

The research began by posing the following questions:

What are the problems of conducting the CCE for final-year nursing students during COVID-19?

How can these problems be addressed?

What are the solutions and suggestions from the involved stakeholders?

How can the CCE be designed, implemented, and evaluated?

What is the impact of exam type on student anxiety and satisfaction?

These questions guided the research in exploring the complexities of administering the CCE amidst the COVID-19 pandemic and in devising practical solutions to ensure the validity and reliability of the assessment while meeting stakeholders’ needs.

## Materials and methods

### Research setting, expert panel members, job analysis, and role delineation

This action research was conducted at the Nursing and Midwifery School of Lorestan University of Medical Sciences, with a history of approximately 40 years. The school accommodates 500 undergraduate and graduate nursing students across six specialized fields, with 84 students enrolled in their final year of undergraduate studies. Additionally, the school employs 26 full-time faculty members in nursing education departments.

An expert panel was assembled, consisting of faculty members specializing in various areas, including medical-surgical nursing, psychiatric nursing, community health nursing, pediatric nursing, and intensive care nursing. The panel also included educational department managers and the examination department supervisor. Through focused group discussions, the panel identified and examined issues regarding the exam format, and members proposed various solutions. Subsequently, after analyzing the proposed solutions and drawing upon the panel members’ experiences, specific roles for each member were delineated.

### Sampling and participant selection

Given the nature of the research, purposive sampling was employed, ensuring that all individuals involved in the design, implementation, and evaluation of the exam participated in this study.

The participants in this study included final-year nursing students, faculty members, clinical skills center experts, the dean of the school, the educational deputy, group managers, and the exam department head. However, in the outcome evaluation phase, 13 faculty members participated in-person and virtually (26 times), and 84 final-year nursing students enrolled in the study using a census method in two semesters of 2020–2021 completed the questionnaires, including 37 females and 47 males. In addition, three male and ten female faculty members participated in this study; of this number, 2 were instructors, and 11 were assistant professors.

### Data collection tools

In order to enhance the validity and credibility of the study and thoroughly examine the results, this study utilized a triangulation method consisting of demographic information, focus group discussions, the Spielberger Anxiety Scale questionnaire, and an Acceptance and Satisfaction Questionnaire.

### Demographic information

A questionnaire was used to gather demographic information from both students and faculty members. For students, this included age, gender, and place of residence, while for faculty members, it included age, gender, field of study, and employment status.

### Focus group discussion

Multiple focused group discussions were conducted with the participation of professors, administrators, experts, and students. These discussions were held through various platforms such as WhatsApp Skype, and in-person meetings while adhering to health protocols. The researcher guided the talks toward the research objectives and raised fundamental questions, such as describing the strengths and weaknesses of the previous exam, determining how to conduct the CCE considering the COVID-19 situation, deciding on virtual and in-person stations, specifying the evaluation checklists for stations, and explaining the weighting and scoring of each station.

### Spielberger anxiety scale questionnaire

This study used the Spielberger Anxiety Questionnaire to measure students’ overt and covert anxiety levels. This questionnaire is an internationally standardized tool known as the STAI questionnaire that measures both overt (state) and covert (trait) anxiety [22]. The state anxiety scale (Form Y-1 of STAI) comprises twenty statements that assess the individual’s feelings at the moment of responding. The trait anxiety scale (Form Y-2 of STAI) also includes twenty statements that measure individuals’ general and typical feelings. The scores of each of the two scales ranged from 20 to 80 in the current study. The reliability coefficient of the test for the apparent and hidden anxiety scales, based on Cronbach’s alpha, was confirmed to be 0.9084 and 0.9025, respectively [23, 24]. Furthermore, in the present study, Cronbach’s alpha value for the total anxiety questionnaire, overt anxiety, and covert anxiety scales were 0.935, 0.921, and 0.760, respectively.

### Acceptance and satisfaction questionnaire

The Acceptability and Satisfaction Questionnaire for Clinical Competency Test was developed by Farajpour et al. (2012). The student questionnaire consists of ten questions, and the professor questionnaire consists of eleven questions, using a four-point Likert scale. Experts have confirmed the validity of these questionnaires, and their Cronbach’s alpha coefficients have been determined to be 0.85 and 0.87 for the professor and student questionnaires, respectively [6]. In the current study, ten medical education experts also confirmed the validity of the questionnaires. Regarding internal reliability, Cronbach’s alpha coefficients for the student satisfaction questionnaire for both virtual and in-person sections were 0.76 and 0.87, respectively. The professor satisfaction questionnaires were 0.84 and 0.87, respectively. An online platform was used to collect data for the virtual exam.

### Data analysis and rigor of study

Qualitative data analysis was conducted using the method proposed by Graneheim and Lundman. Additionally, the criteria established by Lincoln and Guba (1985) were employed to confirm the rigor and validity of the data, including credibility, transferability, dependability, and confirmability [26].

In this research, data synthesis was performed by combining the collected data with various tools and methods. The findings of this study were reviewed and confirmed by participants, supervisors, mentors, and experts in qualitative research, reflecting their opinions on the alignment of findings with their experiences and perspectives on clinical competence examinations. Therefore, the member check method was used to validate credibility.

Moreover, efforts were made in this study to provide a comprehensive description of the research steps, create a suitable context for implementation, assess the views of others, and ensure the transferability of the results.

Furthermore, researchers’ interest in identifying and describing problems, reflecting, designing, implementing, and evaluating clinical competence examinations, along with the engagement of stakeholders in these examinations, was ensured by the researchers’ long-term engagement of over 25 years with the environment and stakeholders, seeking their opinions and considering their ideas and views. These factors contributed to ensuring confirmability.

In this research, by reflecting the results to the participants and making revisions by the researchers, problem clarification and solution presentation, design, implementation, and evaluation of operational programs with stakeholder participation and continuous presence were attempted to prevent biases, assumptions, and research hypotheses, and to confirm dependability.

Data analysis was performed using SPSS version 21, and descriptive statistical tests (absolute and relative frequency, mean, and standard deviation) and inferential tests (paired t-test, independent t-test, and analysis of variance) were used. The significance level was set at 0.05. Parametric tests were used based on the normality of the data according to the Kolmogorov-Smirnov statistical test.

### Study type

Given that conducting the CCE for final-year nursing students required the active participation of managers, faculty members, staff, and students, and to answer the research question “How can the CCE for final-year nursing students be conducted?” and achieve the research objective of “designing, implementing, and evaluating the clinical competency exam,” the action research method was employed.

**Fig. 1:**
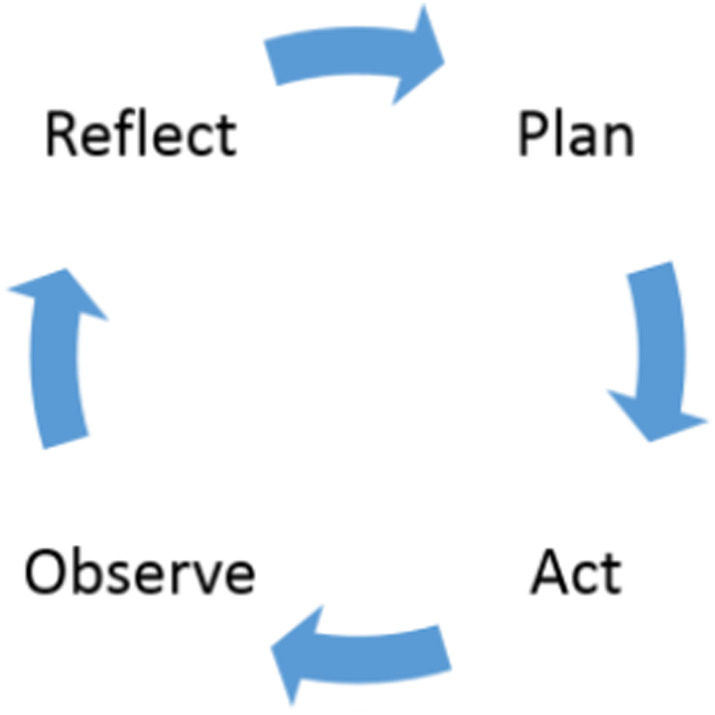
The cyclical process of action research [27]

The present study was conducted based on the Dickens & Watkins model. There are four primary stages (Fig. 1) in the cyclical action research process: reflect, plan, act, observe, and then reflect to continue through the cycle [27].

### Stage 1: Reflection

#### Identification of the problem

According to the educational regulations, final semester nursing students must complete the clinical competency exam. However, due to the COVID-19 pandemic and the critical situation in most provinces, inter-city travel restrictions, and insufficient dormitory space, conducting the CCE in-person was not feasible.

This exam was conducted virtually at our institution. However, based on the reflections from experts, researchers have found that virtual exams can only partially assess clinical and practical skills in certain stations, such as basic skills, resuscitation, and pediatrics. Furthermore, utilizing Objective Structured Clinical Examination (OSCE) in skills assessment facilitates the evaluation of psychomotor skills, knowledge, and attitudes, aiding in identifying strengths and weaknesses.

P3, “Due to the COVID-19 pandemic and the critical situation in most provinces, inter-city travel restrictions, and insufficient dormitory space, conducting the CCE in-person is not feasible.”

### Stage 2: Planning

**Fig. 2:**
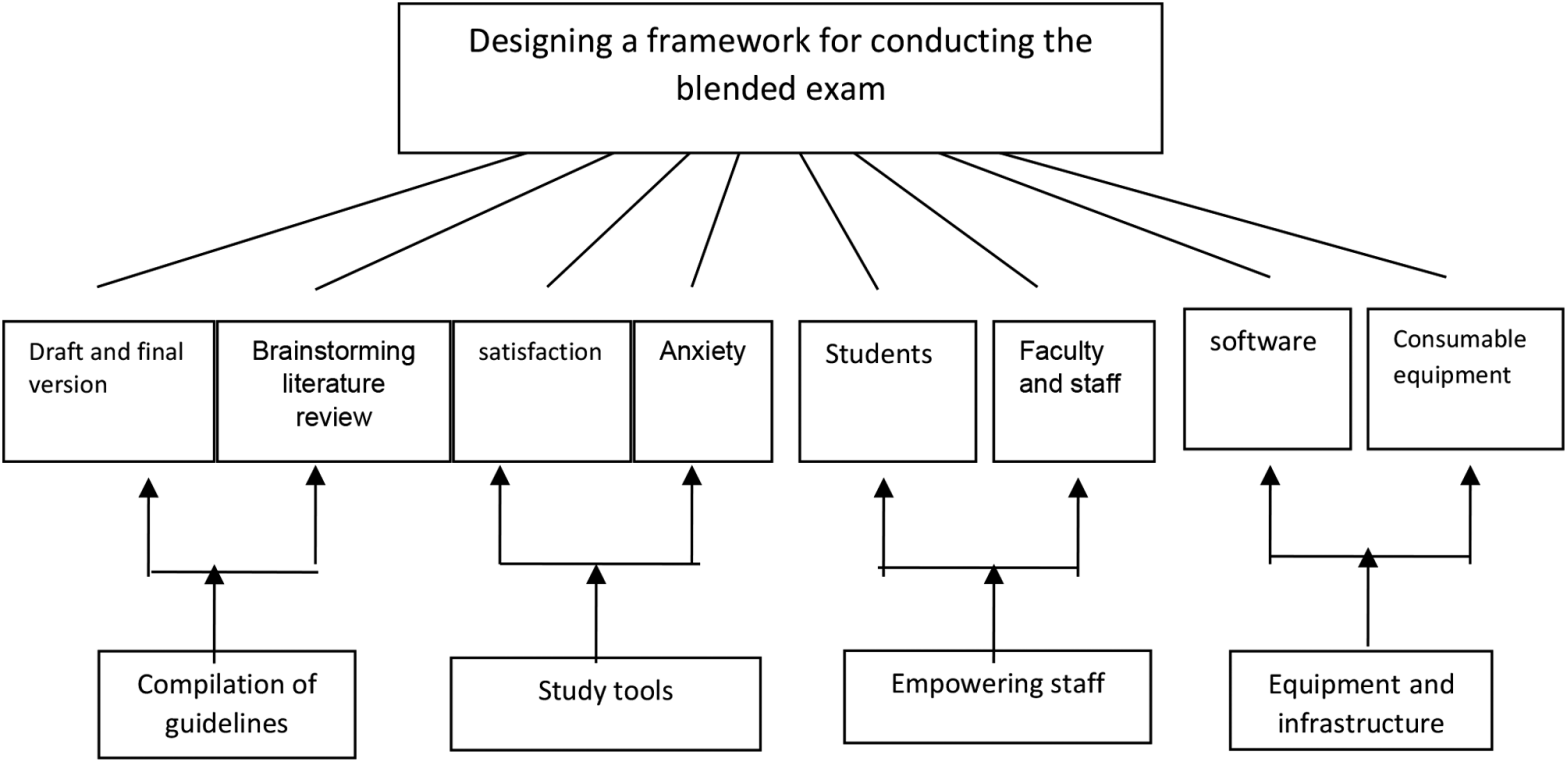
Design and implementation of the blended CCE

Based on the reflections gathered from the participants, the exam was designed using a blended approach (combining in-person and virtual components) as per the schedule outlined in Fig. 2. All planned activities for the blended CCE for final-year nursing students were executed over two semesters.

P5, “Taking the exam virtually might seem easier for us and the students, but in my opinion, it’s not realistic. For instance, performing wound dressing or airway management is very practical, and it’s not possible to assess students with a virtual scenario. We need to see them in person.”

P6"I believe it’s better to conduct those activities that are highly practical in person, but for those involving communication skills like report writing, professional ethics, etc., we can opt for virtual assessment.”

### Stage 3: Act

#### CCE implementation steps

**Fig. 3:**
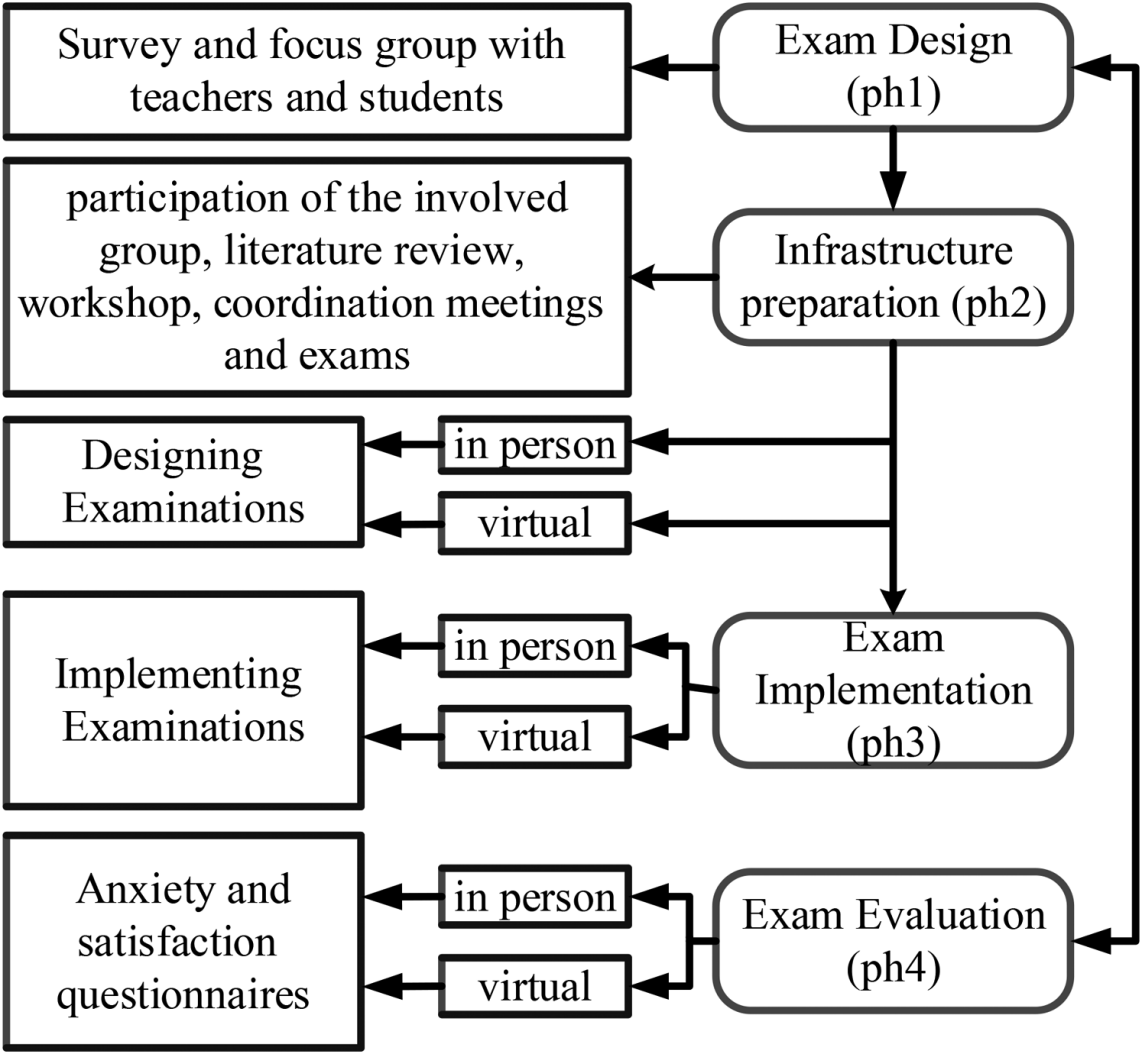
Steps for conducting the CCE for final-year nursing students using a blended method

The CCE was conducted based on the flowchart in Fig. 3 and the following steps:

### Step 1: Designing the framework for conducting the blended Clinical Competency Examination

The panelists were guided to design the blended exam in focused group sessions and virtual panels based on the ADDIE (Analysis, Design, Development, Implementation, Evaluation) model [28]. Initially, needs assessment and opinion polling were conducted, followed by the operational planning of the exam, including the design of the blueprint table (Table 1), determination of station types (in-person or virtual), designing question stems in the form of scenarios, creating checklists and station procedure guides by expert panel groups based on participant analysis, and the development of exam implementation guidelines with participant input [27]. The design, execution, and evaluation were as follows:

1. In-person and virtual meetings with professors were held to determine the exam schedule, deadlines for submitting checklists, decision-making regarding the virtual or in-person nature of stations based on the type of skill (practical, communication), and presenting problems and solutions. Based on the decisions, primary skill stations, as well as cardiac and pediatric resuscitation stations, were held in person. In contrast, virtual stations for health, nursing ethics, nursing reports, nursing diagnosis, physical examinations, and psychiatric nursing were held.
2. News about the exam was communicated to students through the college website and text messages. Then, an online orientation session was held on Skype with students regarding the need assessment of pre-exam educational workshops, virtual and in-person exam standards, how to use exam software, how to conduct virtual exams, explaining the necessary infrastructure for participating in the exam by students, completing anxiety and satisfaction questionnaires, rules and regulations, how to deal with rejected individuals, and exam testing and Q&A. Additionally, a pre-exam in-person orientation session was held.
3. To inform students about the entire educational process, the resources and educational content recommended by the professors, including PDF files, photos and videos, instructions, and links, were shared through a virtual group on the social media messenger, and scientific information was also, questions were asked and answered through this platform.
4. Correspondence and necessary coordination were made with the university clinical skills center to conduct in-person workshops and exams.

Following the Test-centered approach, the Angoff Modified method [29, 30] was used to determine the scoring criteria for each station by panelists tasked with assigning scores.

Additionally, in establishing standards for this blended CCE for fourth-year nursing students, for whom graduation was a prerequisite, the panelists, as experienced clinical educators familiar with the performance and future roles of these students and the assessment method of the blended exam, were involved [29, 30](Table 1).

**Table 1:** Blueprint table of the nursing student Clinical Competency Examination

Station	Percentage (%)
Basic Skills (Dressing and Injections)	15%
Advanced Skills (Cardiopulmonary Resuscitation)	15%
Pediatrics	15%
Psychiatric Nursing	8%
Public Health	8%
Professional Ethics	9%
Nursing notes	10%
Physical Examinations	10%
Nursing Diagnosis	10%

### Step 2: Preparing the necessary infrastructure for conducting the exam

#### Software infrastructure

The pre- and post-virtual exam questions, scenarios, and questionnaires were uploaded using online software.

The exam was conducted on a trial basis in multiple sessions with the participation of several faculty members, and any issues were addressed. Students were authenticated to enter the exam environment via email and personal information verification. The questions for each station were designed and entered into the software by the respective station instructors and the examination coordinator, who facilitated the exam. The questions were formatted as clinical scenarios, images, descriptive questions, and multiple-choice questions, emphasizing the clinical and practical aspects. This software had various features for administering different types of exams and various question formats, including multiple-choice, descriptive, scenario-based, image-based, video-based, matching, Excel output, and graphical and descriptive statistical analyses. It also had automatic questionnaire completion, notification emails, score addition to questionnaires, prevention of multiple answer submissions, and the ability to upload files up to 4 gigabytes. Student authentication was based on national identification numbers and student IDs, serving as user IDs and passwords. Students could enter the exam environment using their email and multi-level personal information verification. If the information did not match, individuals could not access the exam environment.

#### Checklists and questionnaires

A student list was prepared, and checklists for the in-person exam and anxiety and satisfaction questionnaires were reproduced.

#### Empowerment workshops for professors and education staff

Educational needs of faculty members and academic staff include conducting clinical competency exams using the OSCE method; simulating and evaluating OSCE exams; designing standardized questions, checklists, and scenarios; innovative approaches in clinical evaluations; designing physical spaces and setting up stations; and assessing ethics and professional commitment in clinical competency exams.

#### Student empowerment programs

According to the students’ needs assessment results, in-person workshops on cardiopulmonary resuscitation and airway management and online workshops were held on health, pediatrics, cardiopulmonary resuscitation, ethics, nursing diagnosis, and report writing through Skype messenger. In addition, vaccination notes, psychiatric nursing, and educational files on clinical examinations and basic skills were recorded by instructors and made available to students via virtual groups.

### Step 3: CCE implementation

The CCE was held in two parts, in-person and virtual.

#### In-person exam

The OSCE method was used for this section of the exam. The basic skills station exam included dressing and injections, and the CPR and pediatrics stations were conducted in person. The students were divided into two groups of 21 each semester, and the exam was held in two shifts. While adhering to quarantine protocols, the students performed the procedures for seven minutes at each station, and instructors evaluated them using a checklist. An additional minute was allotted for transitioning to the next station.

#### Virtual exam

The professional ethics, nursing diagnosis, nursing report, health, psychiatric nursing, and physical examination stations were conducted virtually after the in-person exam. This exam was made available to students via a primary and a secondary link in a virtual space at the scheduled time. Students were first verified, and after the specified time elapsed, the ability to respond to inactive questions and submitted answers was sent. During the exam, full support was provided by the examination center.

The examination coordinator conducted the entire virtual exam process. The exam results were announced 48 h after the exam. A passing grade was considered to be a score higher than 60% in all stations. Students who failed in various stations were given the opportunity for remediation based on faculty feedback, either through additional study or participation in educational workshops. Subsequent exams were held one week apart from the initial exam. It was stipulated that students who failed in more than half of the stations would be evaluated in the following semester. If they failed in more than three sessions at a station, a decision would be made by the faculty’s educational council. However, no students met these situations.

### Step 4: Evaluation

The evaluation of the exam was conducted by examiners using a checklist, and the results were announced as pass or fail.

### Stage 4: Observation / evaluation

In this study, both process and outcome evaluations were conducted:

#### Process evaluation

All programs and activities implemented during the test design and administration process were evaluated in the process evaluation. This evaluation was based on operational program control and reflections received from participants through group discussion sessions and virtual groups.

Sample reflections received from faculty members, managers, experts, and students through group discussions and social messaging platforms after the changes:

P7: “The implementation of the blended virtual exam, in the conditions of the COVID-19 crisis where the possibility of holding in-person exams was not fully available, in my opinion, was able to improve the quality of exam administration and address the limitations and weaknesses of the exam entirely virtually.”

P5: “In my opinion, this blended method was able to better evaluate students in terms of clinical readiness for entering clinical practice.”

#### Outcomes evaluation

The study outcomes were student anxiety, student acceptance and satisfaction, and faculty acceptance and satisfaction. Before the start of the in-person and virtual exams, the Spielberger Anxiety Questionnaire was provided to students. Additionally, immediately after the exam, students and instructors completed the acceptance and satisfaction questionnaire for the relevant section. After the exam, students and instructors completed the acceptance and satisfaction questionnaire again for the entire exam process, including feasibility, satisfaction with its implementation, and educational impact.

## Findings

### Design framework and implementation for the blended Clinical Competency Examination

The exam was planned using a blended method (part in-person, part virtual) according to the Fig. 2 schedule, and all planned programs for the blended CCE for final-year nursing students were implemented in two semesters.

### Evaluation results

In this study, 84 final-year nursing students participated, including 37 females (44.05%) and 47 males (55.95%). Among them, 28 (33.3%) were dormitory residents, and 56 (66.7%) were non-dormitory residents.

In this study, both process and outcome evaluations were conducted.

#### Process evaluation

All programs and activities implemented during the test design and administration process were evaluated in the process evaluation (Table 2). This evaluation was based on operational program control and reflections received from participants through group discussion sessions and virtual groups on social media.

**Table 2:** Reflections received from faculty, managers, experts, and students via group discussions and social messaging apps following the implementation of changes

Participants’ Statements	Data and Subcategories (Effectiveness)	Initial Coding
P2 (Faculty member): “The blended in-person-virtual exam seems much better than fully virtual because, due to the COVID-19 conditions, we cannot have all stations in person. Last term, we were forced to conduct the entire exam virtually, but in my opinion, taking some stations that were highly clinical in-person makes it better and closer to reality.”	The blended in-person-virtual exam is closer to reality compared to the entirely virtual.	The blended in-person-virtual exam was closer to reality.
P1 (Managers and Specialists): “Compared to the in-person exam, costs and the need for human resources decreased.”	Reducing costs Reducing the need for human resources	We are reducing costs and human resources.
P11(Managers and Specialists): “The severe shortage of nursing staff was largely resolved with the graduation of students, as most are native to the province.”	Solving the severe shortage of nursing staff in the province	Staffing nursing personnel in the province.
P17 (Student): “I’m satisfied that the exam was conducted in two stages. We had less physical presence, and there was no need to stay in quarantine for a long time due to these COVID-19 conditions. Thankfully, none of us showed any symptoms after the exam.”	Satisfaction with the two-stage exam Reduced physical presence Less time in quarantine No COVID symptoms post-exam	Satisfaction with the two-stage exam Reduced physical presence Satisfaction with shorter quarantine No post-exam COVID symptoms

#### Outcomes evaluation

Anxiety and satisfaction were examined and evaluated as study outcomes, and the results are presented below.

The paired t-test results in Table 3 showed no statistically significant difference in overt anxiety (*p* = 0.56), covert anxiety (*p* = 0.13), and total anxiety scores (*p* = 0.167) between the in-person and virtual sections before the blended Clinical Competency Examination.

**Table 3:** Comparison of the mean (SD) total, overt, and covert anxiety scores of final-year nursing students in-person and virtual sections before the blended Clinical Competency Examination

Variable	section	Number	Mean (SD)	statistic test (t)	P*
Overt anxiety	Virtual	84	48.03(11.15)	0.58	0.56
	in person	84	46.84(12.60)		
Covert anxiety	Virtual	84	41.63(8.20)	1.25	0.13
	in person	84	39.69(12.60)		
Total anxiety	Virtual	84	89.57(15.65)	1.39	0.167
	in person	84	85.45(19.76)		

*Paired T-test

However, the mean (SD) of overt anxiety in persons in males and females was 49.27 (11.16) and 43.63 (13.60), respectively, and this difference was statistically significant (*p* = 0.03). Also, the mean (SD) of overt virtual anxiety in males and females was 45.70 (11.88) and 51.00 (9.51), respectively, and this difference was statistically significant (*p* = 0.03). However, there was no significant difference between males and females regarding covert anxiety in the person (*p* = 0.94) and virtual (*p* = 0.60) sections. In addition, the highest percentage of overt anxiety was apparent in the virtual section among women (15.40%) and the in-person section among men (21.28%) and was prevalent at a moderate to high level.

According to Table 4, One-way analysis of variance showed a significant difference between the virtual, in-person, and blended sections in terms of acceptance and satisfaction scores.

The results of the One-way analysis of variance showed that the mean (SD) acceptance and satisfaction scores of nursing students of the CCE in virtual, in-person, and blended sections were 25.49 (4.73), 27.60 (4.70), and 25.57 (4.97) out of 30, respectively. There was a significant difference between the three sections (*p* = 0.008).

**Table 4:** Comparison of the mean (SD) acceptance and satisfaction scores of nursing students from the CCE in person, virtual, and blended sections

Acceptance and satisfaction	Section	Number	Mean (SD)	Statistic test (F)	P*
Female student	in person	34	27.60 (4.59)	1.38	0.258
	virtual	34	25.97 (4.66)		
	blended	34	25.52 (5.20)		
Male student	in person	45	27.84 (4.75)	3.88	0.024
	virtual	45	25.13 (4.80)		
	blended	45	25.60 (4.89)		
Total student	in person	79	27.60 (4.70)	-2.70	0.008
	virtual	79	25.49 (4.73)		
	blended	79	25.57 (4.97)		

* One-way ANOVA

In addition, 3 (7.23%) male and 10 (76.3%) female faculty members participated in this study; of this number, 2 (15.38%) were instructors, and 11 (84.62%) were assistant professors. Moreover, they were between 29 and 50 years old, with a mean (SD) of 41.37 (6.27). Furthermore, they had 4 to 20 years of work experience with a mean and standard deviation of 13.22(4.43).

The results of the analysis of variance showed that the mean (SD) acceptance and satisfaction scores of faculty members of the CCE in virtual, in-person, and blended sections were 30.31 (4.47), 29.86 (3.94), and 30.00 (4.16) out of 33, respectively. There was no significant difference between the three sections (*p* = 0.864).

## Discussion

This action research study showed that the blended CCE for nursing students is feasible and, depending on the conditions and objectives, evaluation stations can be designed and implemented virtually or in person.

The blended exam, combining in-person and virtual elements, managed to address some of the weaknesses of entirely virtual exams conducted in previous terms due to the COVID-19 pandemic. Given the pandemic conditions, the possibility of performing all in-person stations was not feasible due to the risk of students and evaluators contracting the virus, as well as the need for prolonged quarantine. Additionally, to meet the staffing needs of hospitals, nursing students needed to graduate. By implementing the blended exam idea and conducting in-person evaluations at clinical stations, the assessment of nursing students’ clinical competence was brought closer to reality compared to the entirely virtual method.

Furthermore, the need for human resources, station setup costs, and time spent was less than the entirely in-person method. Therefore, in pandemics or conditions where sufficient financial resources and human resources are not available, the blended approach can be utilized.

Additionally, the evaluation results showed that students’ total and overt anxiety in both virtual and in-person sections of the blended CCE did not differ significantly. However, the overt anxiety of female students in the virtual section and male students in the in-person section was considerably higher. Nevertheless, students’ covert anxiety related to personal characteristics did not differ in virtual and in-person exam sections. However, students’ acceptance and satisfaction in the in-person section were higher than in the virtual and blended sections, with a significant difference. The acceptance and satisfaction of faculty members from the CCE in in-person, virtual, and blended sections were the same and relatively high.

A blended CCE nursing competency exam was not found in the literature review. However, recent studies, especially during the COVID-19 pandemic, have designed and implemented this exam using virtual OSCE. Previously, the CCE was held in-person or through traditional OSCE methods.

During the COVID-19 pandemic, nursing schools worldwide faced difficulties administering clinical competency exams for students. The virtual simulation was used to evaluate clinical competency and develop nursing students’ clinical skills in the United States, including standard videos, home videos, and clinical scenarios. Additionally, an online virtual simulation program was designed to assess the clinical competency of senior nursing students in Hong Kong as a potential alternative to traditional clinical training [31].

A traditional in-person OSCE was also redesigned and developed through a virtual conferencing platform for nursing students at the University of Texas Medical Branch in Galveston. Survey findings showed that most professors and students considered virtual OSCE a highly effective tool for evaluating communication skills, obtaining a medical history, making differential diagnoses, and managing patients. However, professors noted that evaluating examination techniques in a virtual environment is challenging [32].

However, Biranvand reported that less than half of the nursing students believed the in-person OSCE was stressful [33]. At the same time, the results of another study showed that 96.2% of nursing students perceived the exam as anxiety-provoking [1]. Students believe that the stress of this exam is primarily related to exam time, complexity, and the execution of techniques, as well as confusion about exam methods [7]. In contrast to previous research results, in a study conducted in Egypt, 75% of students reported that the OSCE method has less stress than other examination methods [9]. However, there has yet to be a consensus across studies on the causes and extent of anxiety-provoking in the OSCE exam. In a study, the researchers found that in addition to the factors mentioned above, the evaluator’s presence could also be a cause of stress [34]. Another survey study showed that students perceived the OSCE method as more stressful than the traditional method, mainly due to the large number of stations, exam items, and time constraints [7]. Another study in Egypt, which designed two stages of the OSCE exam for 75 nursing students, found that 65.6% of students reported that the second stage exam was stressful due to the problem-solving station. In contrast, only 38.9% of participants considered the first-stage exam stressful [35]. Given that various studies have reported anxiety as one of the disadvantages of the OSCE exam, in this study, one of the outcomes evaluated was the anxiety of final-year nursing students. There was no significant difference in total anxiety and overt anxiety between students in the in-person and virtual sections of the blended Clinical Competency Examination. The overt anxiety was higher in male students in the in-person part and female students in the virtual section, which may be due to their personality traits, but further research is needed to confirm this. Moreover, since students’ total and overt anxiety in the in-person and virtual sections of the exam are the same in resource and workforce shortages or pandemics, the blended CCE is suggested as a suitable alternative to the traditional OSCE test. However, for generalization of the results, it is recommended that future studies consider three intervention groups, where all OSCE stations are conducted virtually in the first group, in-person in the second group, and a blend of in-person and virtual in the third group. Furthermore, the results of the study by Rafati et al. showed that the use of the OSCE clinical competency exam using the OSCE method is acceptable, valid, and reliable for assessing nursing skills, as 50% of the students were delighted, and 34.6% were relatively satisfied with the OSCE clinical competency exam. Additionally, 57.7% of the students believed the exam revealed learning weaknesses [1]. Another survey study showed that despite higher anxiety about the OSCE exam, students thought that this exam provides equal opportunities for everyone, is less complicated than the traditional method, and encourages the active participation of students [7]. In another study on maternal and infant care, 95% of the students believed the traditional exam only evaluates memory or practical skills. In contrast, the OSCE exam assesses knowledge, understanding, cognitive and analytical skills, communication, and emotional skills. They believed that explicit evaluation goals, appropriate implementation guidelines, appropriate scheduling, wearing uniforms, equipping the workroom, evaluating many skills, and providing fast feedback are among the advantages of this exam [36]. Moreover, in a survey study, most students were satisfied with the clinical environment offered by the OSCE CCE using the OSCE method, which is close to reality and involves a hypothetical patient in necessary situations that increase work safety. On the other hand, factors such as the scheduling of stations and time constraints have led to dissatisfaction among students [37].

Furthermore, another study showed that virtual simulations effectively improve students’ skills in tracheostomy suctioning, triage concepts, evaluation, life-saving interventions, clinical reasoning skills, clinical judgment skills, intravenous catheterization skills, role-based nursing care, individual readiness, critical thinking, reducing anxiety levels, and increasing confidence in the laboratory, clinical nursing education, interactive communication, and health evaluation skills. In addition to knowledge and skills, new findings indicate that virtual simulations can increase confidence, change attitudes and behaviors, and be an innovative, flexible, and hopeful approach for new nurses and nursing students [38].

Various studies have evaluated the satisfaction of students and faculty members with the OSCE Clinical Competency Examination. In this study, one of the evaluated outcomes was the acceptability and satisfaction of students and faculty members with implementing the CCE in blended, virtual, and in-person sections, which was relatively high and consistent with other studies. One crucial factor that influenced the satisfaction of this study was the provision of virtual justification sessions for students and coordination sessions with faculty members. Social messaging groups were formed through virtual and in-person communication, instructions were explained, expectations and tasks were clarified, and questions were answered. Students and faculty members could access the required information with minimal presence in medical education centers and time and cost constraints. Moreover, with the blended evaluation, the researcher’s communication with participants was more accessible. The written guidelines and uploaded educational content of the workshops enabled students to save the desired topics and review them later if needed. Students had easy access to scientific and up-to-date information, and the application of social messengers and Skype allowed for sending photos and videos, conducting workshops, and questions and answering questions. However, the clinical workshops and examinations were held in-person to ensure accuracy. The virtual part of the examination was conducted through online software, and questions focused on each station’s clinical and practical aspects. Students answered various questions, including multiple-choice, descriptive, scenario, picture, and puzzle questions, within a specified time. The blended examination evaluated clinical competency and did not delay these individuals’ entry into the job market. Moreover, during the severe human resource shortage faced by the healthcare system, the examination allowed several nurses to enter the country’s healthcare system. The blended examination can substitute in-person examination in pandemic and non-pandemic situations, saving facilities, equipment, and human resources. The results of this study can also serve as a model to guide other nursing departments that require appropriate planning and arrangements for Conducting Clinical Competency Examinations in blended formats. This examination can also be developed to evaluate students’ clinical performance.

One of the practical limitations of the study was the possibility that participants might need to complete the questionnaires accurately or be concerned about losing marks. Therefore, in a virtual session before the in-person exam, the objectives and importance of the study were explained. Participants were assured that it would not affect their evaluation and that they should not worry about losing marks. Additionally, active participation from all nursing students, faculty members, and staff was necessary for implementing this plan, achieved through prior coordination, virtual meetings, virtual group formation, and continuous reflection of results, creating the motivation for continued collaboration and participation.

Among other limitations of this study included the use of the Spielberger Anxiety Questionnaire to measure students’ anxiety. It is suggested that future studies use a dedicated anxiety questionnaire designed explicitly for pre-exam anxiety measurement. Another limitation of the current research was its implementation in nursing and midwifery faculty. Therefore, it is recommended that similar studies be conducted in nursing and midwifery faculties of other universities, as well as in related fields, and over multiple consecutive semesters. Additionally, for more precise effectiveness assessment, intervention studies in three separate virtual, in-person, and hybrid groups using electronic checklists are proposed. Furthermore, it is recommended that students be evaluated in terms of other dimensions and variables such as awareness, clinical skill acquisition, self-confidence, and self-efficacy.

## Conclusion

Conducting in-person Clinical Competency Examination (CCE) during critical situations, such as the COVID-19 pandemic, is challenging. Instead of virtual exams, blended evaluation is a feasible approach to overcome the shortages of virtual ones and closely mimic in-person scenarios. Using a blended method in pandemics or resource shortages, it is possible to design, implement, and evaluate stations that evaluate basic and advanced clinical skills in in-person section, as well as stations that focus on communication, reporting, nursing diagnosis, professional ethics, mental health, and community health based on scenarios in a virtual section, and replace traditional OSCE exams. Furthermore, the use of patient simulators, virtual reality, virtual practice, and the development of virtual and in-person training infrastructure to improve the quality of clinical education and evaluation and obtain the necessary clinical competencies for students is recommended. Also, since few studies have been conducted using the blended method, it is suggested that future research be conducted in three intervention groups, over longer semesters, based on clinical evaluation models and influential on other outcomes such as awareness and clinical skill acquisition self-efficacy, confidence, obtained grades, and estimation of material and human resources costs. This approach reduced the need for physical space for in-person exams, ensuring participant quarantine and health safety with higher quality. Additionally, a more accurate assessment of nursing students’ practical abilities was achieved compared to a solely virtual exam.

## Data Availability

The datasets generated and analyzed during the current study are available on request from the corresponding author.
